# 7-Pyrrolidinethoxy-4′-Methoxyisoflavone Prevents Amyloid β–Induced Injury by Regulating Histamine H3 Receptor-Mediated cAMP/CREB and AKT/GSK3β Pathways

**DOI:** 10.3389/fnins.2019.00334

**Published:** 2019-04-10

**Authors:** Linlin Wang, Jiansong Fang, Hailun Jiang, Qian Wang, Situ Xue, Zhuorong Li, Rui Liu

**Affiliations:** ^1^Institute of Medicinal Biotechnology, Chinese Academy of Medical Sciences & Peking Union Medical College, Beijing, China; ^2^Institute of Materia Medica, Chinese Academy of Medical Sciences & Peking Union Medical College, Beijing, China; ^3^Institute of Clinical Pharmacology, Guangzhou University of Chinese Medicine, Guangzhou, China

**Keywords:** acetylcholine, Alzheimer’s disease, amyloid beta-peptide, cyclic AMP response element binding protein, histamine H3 receptor

## Abstract

In studies on the treatment of Alzheimer’s disease (AD), in which cognition is enhanced even modestly or selectively, it has been considered that the histamine H3 receptor (H3R) may be a potential target. In this study, we aimed at evaluating the ability of 7-pyrrolidinethoxy-4′-methoxyisoflavone (indicated as LC1405), a novel potential H3R antagonist identified from our H3R antagonist screening system, to ameliorate amyloid β (Aβ)-induced cognitive deficits, and to explore the underlying mechanisms that are related to H3R-modulated signaling. Our results demonstrated that LC1405 effectively reduced the progression of Aβ-associated disorders, such as improved learning and memory capabilities, preserved tissues from suffering neurodegeneration and ultrastructural abnormalities, and ameliorated cholinergic dysfunction in an APP/PS1 double transgenic mouse model of AD. In an *in vitro* model, LC1405 protected neuronal cells against copper-induced Aβ toxicity, as demonstrated by the improvement in cell viability and decrease in neuronal apoptotic ratio. In addition, treatment with LC1405 resulted in the up-regulation of acetylcholine (ACh) or histamine release and provided neuroprotection through cellular signaling cascades involving H3R-mediated cAMP/CREB and AKT/GSK3β pathways. Furthermore, the beneficial effects of LC1405 on Aβ-mediated toxicity and H3R-mediated cAMP/CREB and AKT/GSK3β axes were reversed after pharmacological activation of H3R. In conclusion, our results demonstrated that LC1405 blocked Aβ-induced toxicity through H3R-modulated signaling transduction both *in vitro* and *in vivo*. The results also suggested that LC1405 might have translational potential as a complementary therapy to control disease progression in AD patients who developed cognitive deficits with H3R-related ACh neurotransmission abnormality.

## Introduction

Alzheimer’s disease (AD), the most common cause of dementia in elderly population, is characterized by complicated and multifactorial pathophysiological alterations, primarily including senile plaque deposits, Tau protein hyperphosphorylation, high oxidative stress, metal ion dyshomeostasis, and neurotransmitter system irregularities (Ballard et al., 2011; Chiang and Koo, 2014). Currently, the principal treatment to combat AD in clinical practice involves the administration of acetylcholinesterase (AChE) inhibitors and the *N*-methyl-D-aspartate (NMDA) receptor antagonist memantine, which resulted in limited symptomatic improvement. However no effective treatment strategy is available that results in recovery or even retardation in the progression of the disease. Therefore, it is of utmost importance to develop novel and effective therapies for the treatment of AD.

Over the past decade, preclinical studies and clinical trials identified histamine H3 receptor (H3R), a histamine receptor subtype that is predominantly expressed in neurons of the central nervous system (CNS), as a possible target for cognition-enhancing candidates that may have beneficial effects on mild-to-moderate AD (Esbenshade et al., 2006; Bonaventure et al., 2007; Mani et al., 2017). Among the diverse H3R antagonists investigated up to now, two small H3R antagonists, ABT-239 and A-431404, showed procognitive effects in ketamine and MK-801-induced animal models (Brown et al., 2013). Pre-clinical studies demonstrated that several H3R antagonists ameliorate cognitive deficits and related behaviors in a substantial number of animal models characterized by learning or memory dysfunction (Browman et al., 2004).

In AD, H3R in the prefrontal cortex and hippocampus acts as a presynaptic auto-receptor coupled to Gα_i/o_-proteins that controls the synthesis and release of histamine, and as a heteroreceptor on histaminergic and non-histaminergic neurons in regulating the release of other neurotransmitters, including acetylcholine (ACh), dopamine, glutamate, norepinephrine, and gamma-aminobutyric acid (GABA) (Clapham and Kilpatrick, 1992; Ligneau et al., 1998; Bacciottini et al., 2002; Medhurst et al., 2007). Furthermore, within the neuronal intracellular signal transduction H3R participates in a variety of pathways (Clapham and Kilpatrick, 1992; Esbenshade et al., 2008; Bhowmik et al., 2014). When ligands bind to H3R-activated Gi proteins, adenylyl cyclase (AC) is inhibited, leading to decreased levels of cyclic adenosine monophosphate (cAMP) and reduced phosphorylation-activation of cAMP response element binding protein (CREB), a transcription factor that is closely related to cognitive functions (Bhowmik et al., 2014). In addition, pathological alterations of phosphatidylinositol-3-kinase/protein kinase B/glycogen synthase kinase 3β (PI3K/AKT/GSK3β) signaling transduction are due to β-amyloid (Aβ)-stimuli via a variety of signal transduction pathways, thereby amplifying Aβ-induced pathogenic responses in the brain through abnormal H3R agonistic reactions (Bhowmik et al., 2014). Accordingly, a large number of previous studies demonstrated that targeted activation of H3R in neurons resulted in an accelerated cognitive decline and aggravated Aβ-induced neuronal perturbation in β-amyloid precursor protein (APP) transgenic mice, accompanied by progressive loss of cholinergic neurons and destructive signaling pathways involving cAMP/CREB and AKT/GSK3β cascades (Bhowmik et al., 2014; Bardgett et al., 2011; Bitner et al., 2011). Therefore, the central role of H3R in AD suggests that it may be an attractive target in the development of novel therapies against diseases using H3R antagonists.

**FIGURE 1:**
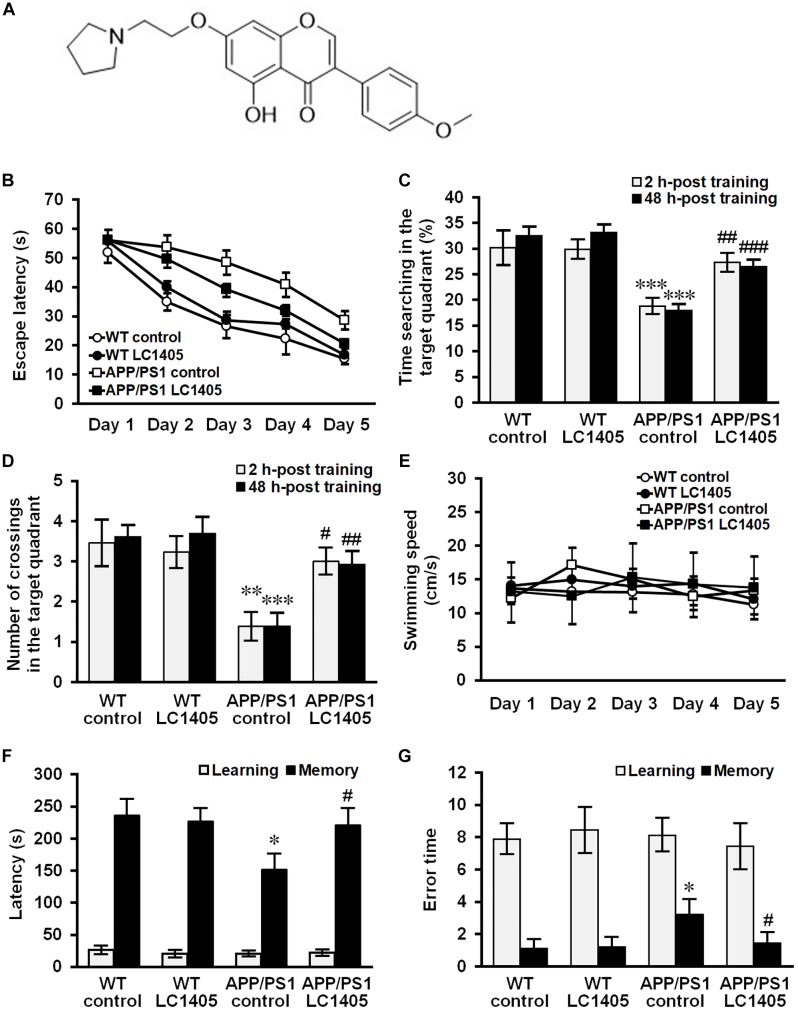
Long-term LC1405 treatment ameliorates cognitive deficits in APP/PS1 mice. **(A)** Chemical structure of LC1405. **(B)** Comparison of latency using the platform Morris Water Maze test during 5 training days. **(C)** LC1405 treatment increased the time mice spent in the target quadrant as determined by the probe test. **(D)** LC1405 treatment increased the number of crossings where the platform was previously located. **(E)** No significant differences in motor function were observed in LC1405-treated mice and vehicle-treated controls as indicated by the swimming speed. **(F)** In the retention trial of the step-through passive avoidance test, LC1405 treatment increased the latency to enter the dark compartment. **(G)** LC1405 treatment resulted in fewer occasions of re-entering the illuminated compartment as a mistake. Data are presented as mean ± SEM, *n* = 13. ^∗^*p* < 0.05, ^∗∗^*p* < 0.01, *^∗∗∗^p* < 0.001 vs. WT control,^#^*p* < 0.05, ^##^*p* < 0.01, ^###^*p* < 0.001 vs. APP/PS1 control.

In the present study, we have investigated cognitive improvement and neuronal protection using a potential non-imidazole H3R antagonist, 7-pyrrolidinethoxy-4′-methoxyisoflavone (Figure 1A, indicated as LC1405) that has affinity with human H3R and high H3R inhibition power *in vitro*. We investigated its action against Aβ-induced neurotoxicity, and the underlying mechanisms of action against Aβ toxicity correlated with H3R-modulated signaling both in APP and presenilin 1 (PS1) double transgenic mice and copper-induced Aβ toxicity in APP Swedish mutation overexpressing SH-SY5Y cells.

## Materials and Methods

### Animals and Drug Treatment

Heterozygous APPswe695/PSEN1dE9 (APP/PS1) transgenic mice and age-matched wild-type (WT, C57BL/6) littermates were obtained from the Model Animal Research Center of Nanjing University (Nanjing, China). All animal studies were approved by the Institute of Medicinal Biotechnology, Chinese Academy of Medical Sciences (Beijing, China) and performed in accordance with ethical guidelines of the Experimental Animal Care and Use Committee.

Nine-month-old WT mice and APP/PS1 transgenic mice were randomly divided into four groups, including a WT control group (*n* = 13, seven males and six females), LC1405-treated WT group (*n* = 13, seven males and six females), APP/PS1 control group (*n* = 13, seven males and six females), and LC1405-treated APP/PS1 group (*n* = 13, seven males and six females). Mice in the WT+LC1405 and APP/PS1+LC1405 groups received an intragastric administration of LC1405 for 6 d/week at a dosage of 3 mg/kg. LC1405 was dissolved in 20% hydroxypropyl-β-cyclodextrin (CMC-Na), thus, mice in the control group received 20% CMC-Na according to the same modality. Drug treatment was performed for 20 weeks.

After behavioral tests, mice were divided into three groups for the parallel evaluation of different parameters. Three mice per group were transcardially perfused with normal saline solution, followed by 4% paraformaldehyde (PFA). Then, brains were collected and subjected to immunohistochemical staining and transmission electron microscopy. Four mice per group were chosen for the detection of oxidative stress, cholinergic activity, Aβ levels, and cAMP content in the brain. Four mice per group were prepared for quantification of H3R-mediated signaling transduction in the cortex and hippocampus, such as phosphorylated CREB, AKT, and GSK3β using ELISA. The brain of the remaining mice was quickly removed and stored at -80°C until further experiments. The acetylcholine and histamine levels were evaluated in another two WT mouse groups (*n* = 4, two males and two females) that received an intragastric administration of LC1405 at a dosage of 3 mg/kg dissolved in CMC-Na or only CMC-Na as control. Mice were randomly selected for both the initial division into treatment groups and the subsequent selection for the three experiments.

### Behavioral Assessment of Learning and Memory

Behavioral tests were performed when mice were thirteen months old, following 20 weeks of treatment with LC1405 or vehicle control. The Morris Water Maze (MWM) test is a classical visual-spatial learning technique used in rodents for assessing learning and memory capabilities hippocampus-dependent (Vorhees and Williams, 2006). Briefly, during the acquisition trial, mice were subjected to four training trials per day for five consecutive days. Both the escape latency (time required to find the platform) and swimming speed were recorded. At the end of the last trial, the platform was removed to proceed for the probe trial, which was carried out at 2 and 48 h post-training. The time the mice spent in the target quadrant and the frequency of passing through the platform were recorded.

Contextual short-term memory of the mice was assessed using a passive avoidance test (Nasehi et al., 2012). The apparatus consisted of illuminated (bright) and non-illuminated (dark) compartments. During the acquisition trial, mice were initially placed in the bright compartment for a maximum of 300 s, and upon entering the dark compartment received an electric foot shock (0.3 mA, 2 s). As a measure of memory retention, the initial latency of mice to enter the dark compartment and error times were recorded at 24 h after acquisition training.

### Immunohistochemical Staining

The brain was embedded in paraffin and dissected into 8 μM sections. Fluoro-Jade B (FJB, Histochem, Jefferson, AR, United States) and Thioflavin S (Thio S, Sigma, St. Louis, MO, United States) were used to evaluate neuronal degeneration and fibrillar Aβ level in hemi-brain tissue sections, respectively, and were performed using standard histological techniques as previously reported (Liu et al., 2014, 2018). Brain pathological features were evaluated on the sections under a fluorescence microscope (Olympus IX70, Olympus, Tokyo, Japan). The number of FJB-positive neurons and Thio S-positive neurons were manually evaluated as the number of neurons in 1 mm^2^ of the cerebral cortex and hippocampus region. Cell count was obtained by averaging the counts from 10 sections per mouse.

### Ultrastructural Analysis by Transmission Electron Microscopy

The prefrontal cortex and hippocampus were carefully harvested from the PFA-perfused brain of the experimental mice and placed overnight in the fixative [20 mL of 2.5% glutaraldehyde (Merck, Darmstadt, Germany) and 2.0% PFA (Beijing Chemical Works, Beijing, China) in 0.15 M cacodylate buffer (Merck, Darmstadt, Germany)]. Ultrathin sections were cut as previously described (Yu et al., 2015), and a LEO 906 transmission electron microscope (Zeiss, Oberkochen, Germany) was used for ultrastructural imaging.

### Measurement of Oxidative Stress and Cholinergic Activity in the Brain

The hemi-brains of APP/PS1 and WT mice were homogenized via ultrasonication, and centrifuged at 12,000 ×*g* for 10 min at 4°C. The concentration of malondialdehyde (MDA), superoxide dismutase (SOD) and glutathione peroxidase (GSH-Px) within the homogenates was determined using commercial assay kits (Jiancheng Biotech, Nanjing, Jiangsu, China) in accordance with the corresponding manufacturer’s instructions. ACh levels and the AChE activity were determined using the ultrasensitive Amplex@ red ACh/AChE assay kit (Molecular Probes, Paisley, United States) according to the manufacturer’s guidelines.

### Acetylcholine Quantification Using High-Performance Liquid Chromatography

For conventional analysis, brain tissue ACh levels were quantified using high-performance liquid chromatography (HPLC) coupled to electrochemical detection. In brief, ACh was separated on a BetaBasic C18 column (Thermo-Hypersil, Waltham, MA, United States; 150 mm × 1.0 mm; particle size, 3 μm) at a temperature of 25 °C using a mobile phase consisting of 100 mM Na_2_HPO_4_, 2.0 mM sodium octanesulfonic acid, 0.5 mM tetramethylammonium chloride and 100 μL of Reagent MB microbicide (ESA Inc., Chelmsford, MA, United States), adjusting the pH to 8.0. The separated ACh fraction was placed in an enzyme reactor (Bioanalytical Systems) which yielded H_2_O_2_ for the detection with an enzyme-coated glassy carbon electrode with a potential set at 100 mV versus silver/silver chloride (Ag/AgCl). The sensitivity for ACh detection was approximately 0.5 fmol/15 μL of sample. Chromatographic data were collected and quantified by comparison with known standard concentrations.

### Histamine Determination Using High-Performance Liquid Chromatography

Brain histamine levels were determined by HPLC and quantified by fluorometric detection. Brains were homogenized in 3% perchloric acid solution containing 0.5 mM EDTA, and then centrifuged at 5500 ×*g* for 15 min at 4°C. The supernatant was collected and histamine was separated using a Waters Atlantis C18 HPLC column (Milford, MA, United States; 150 mm × 3.0 mm; particle size, 3 μm) with a mobile phase containing 160 mM KH_2_PO_4_, 0.45 mM octanesulfonic acid, 1% methanol and 0.1 mM EDTA (pH 4.5), delivered at 0.5 to 0.7 mL/min. Using a T-piece, the eluent line was connected to the reagent line, through which a 0.02% solution of o-phthaldialdehyde (OPA) was delivered in 0.15 M NaOH at a rate of 0.60 mL/min. The OPA reagent was mixed with the eluent in a mixing coil of metal tubing (outer diameter, 1.1 mm; inner diameter, 0.55 mm; length, 1 m) that enabled the derivatization reaction at room temperature. The fluorescence of the reaction product was measured using a fluorometric detector (Ex/Em, 350 nm/450 nm). Chromatographic data were collected and quantified by comparison with known standard concentrations. The sensitivity for histamine detection was approximately 20 fmol/20 μL of sample.

### Cell Culture and Treatments

To evaluate the neuroprotective effects of LC1405, the Swedish mutant form of the human APP695 gene was stably transfected into SH-SY5Y cells that were purchased from the ATCC (ATCC^®^CRL-2266, Manassas, VA, United States), to establish an AD *in vitro* model (named APPsw cells). In this cell model, copper acts as a promotor for triggering Aβ-mediated neurotoxicity when added as a stimulator into the culture medium (Zhang et al., 2006; Zhao et al., 2013). Cells were cultured in DMEM/F12 medium (Invitrogen, Carlsbad, CA, United States) supplemented with 10% fetal calf serum (Invitrogen, Carlsbad, CA, United States) and incubated at 37°C in a humidified chamber containing 5% CO_2_. The detailed protocol and groups were as follows. Cells were randomly divided into two groups: one group was supplemented with 300 μM copper, whereas the other was not (indicated as control cells). The group supplemented with copper was divided into subgroups based on LC1405 concentrations as follows: 0 μM (copper-treated APPsw cells), 0.03 μM, 0.1 μM, 0.3 μM, 1.0 μM, 3.0 μM, and 10.0 μM. Different concentrations of LC1405 were added at the start of copper-initiated injury, then cells were incubated for 24 h at 37°C. To determine whether H3R inhibition was involved in the neuroprotective effects of LC1405 against Aβ toxicity, the specific agonists of H1 to H4, 2-(3-trifluoromethylphenyl) histamine (FMPH), amthamine dihydrobromide, (*R*)-(α)-(-)-methylhistamine dihydrobromide (RAMH) and VUF-8430 were used. Cells were pretreated with the specific agonists at 1.0 μM for 30 min at 37°C before being treated with LC1405.

### Cell Viability Assay

Viability was determined by 3-(4,5-dimethylthiazol-2-yl)-5-(3-carboxymethoxyphenyl)-2-(4-sulfophenyl)-2*H*-tetrazolium (MTS, Promega, Madison, WI, United States). In brief, cells were incubated for 4 h at 37°C with an appropriate amount of MTS according to the manufacturer’s instructions. The soluble product formazan was detected using a Spark 20M multimode microplate reader (Tecan Group Ltd., Mannedorf, Switzerland) at 490 nm.

### Cell Immunofluorescence Assay

Cell immunofluorescence is a sensitive fluorescence-based multiparametric technology that is used to determine the expression and activity of proteins within a cell. Cells were seeded in a 96-well plate at a density of 8000 cells/well in 200 μL medium/well and subjected to all treatments described in the section 2.8. The apoptotic ratio *in vitro* was determined by simultaneous staining with acridine orange (AO) and ethidium bromide (EB) (Sigma-Aldrich, St. Louis, MO, United States). After treatment with LC1405 at different concentrations, 100 μg/mL AO and 100 μg/mL EB were added to the cells and incubated for 15 min at 37°C. The expression of β-APP, p-CREB, and p-PI3K was quantified using cell immunofluorescence routine procedures as previously described (Liu et al., 2012). The following primary polyclonal rabbit antibodies were used: anti-β-APP [1:500, Cell Signaling Technology (CST), Danvers, MA, United States], anti-p-CREB (Ser133) (1:120, CST), and anti-p-PI3K p85α (Y607) (1:80, Abcam, Cambridge, MA, United States). The fluorescent secondary antibodies used were goat anti-rabbit conjugated with Alexa Fluor 488 or Alexa Fluor 546 (1:1000, Invitrogen, Carlsbad, CA, United States). The apoptotic ratio and mean fluorescence intensity were determined and analyzed using a Cellomics ArrayScan ^V TI^ HCS Reader (Cellomics Inc., Pittsburgh, PA, United States) running Morphology Explorer BioApplication software for the average of 20 fields of view in each selected well.

### Determination of Aβ and cAMP Concentrations

After the treatment, mouse hemi-brains and APPsw cells were separately homogenized using ultrasonication and centrifuged at 12,000 ×*g* for 10 min at 4°C. An aliquot of mouse hemi-brains or APPsw cells after homogenization was resuspended in 2% sodium dodecyl sulfate (SDS) containing protease inhibitors. After centrifugation, the supernatant was collected for the detection of soluble Aβ. The remaining SDS-insoluble pellet was sonicated, dissolved in 70% formic acid, and centrifuged for 60 min at 100,000 ×*g*, and the supernatant was collected for the detection of insoluble Aβ. The concentration of both soluble and insoluble Aβ was quantified using commercially available ELISA kits (BioSource, Camarillo, CA, United States) specific for the detection of human Aβ_1-40/42_. In addition, soluble Aβ oligomers (oAβ) were detected using western blot as previously described (Liu et al., 2018). Another aliquot of mouse hemi-brains was homogenized in RIPA buffer (Cell Signaling Technology, Danvers, MA, United States) containing protease inhibitor, phosphatase inhibitor, and phenylmethyl sulfonylfluoride (PMSF). Forty μg protein per lane were run on polyacrylamide gel, transferred onto a polyvinylidenedifluoride membrane, blocked with 5% BSA in Tris-buffer saline containing 0.1% Tween-20 (TBST) for 2 h, and subsequently incubated with the primary antibody overnight, using the rabbit anti-oligomer conformation-specific A11 (pre-fibrillar Aβ oligomer, 1:1000, Invitrogen, Carlsbad, CA, United States) diluted in blocking solution. Membranes were washed with TBST prior to incubation with horseradish peroxidase-labeled (HRP)-linked secondary antibody (1:1000, ZSGB-Bio, Beijing, China) at room temperature for 1 h. The signals were detected using an enhanced chemiluminescence kit. Chemiluminescence image acquisition and densitometric band quantitation were performed using Fusion-FX6 imaging system (Vilber Lourmat, Marne-la-Valle, France). The cAMP concentration was determined using a cAMP assay kit (R&D Systems, Minneapolis, MN, United States) in accordance with the manufacturer’s guidelines.

### Quantification of Phosphorylated CREB, AKT, and GSK3β Using ELISA

After the treatment, the cortex and hippocampal tissue of APP/PS1 and WT mice and APPsw cells were homogenized after addition of RIPA buffer (CST, Danvers, MA, United States) containing protease inhibitors, phosphatase inhibitors, and PMSF, then centrifuged at 20,000 ×*g* for 15 min at 4°C. The protein concentration of the samples was quantified using a commercially available bicinchoninic acid (BCA) kit (Thermo Fisher Scientific, Rockford, IL, United States). ELISA kits were used for quantification of phospho-CREB (Ser133, R&D Systems, Minneapolis, MN, United States), phospho-AKT (Ser473, R&D Systems) and phospho-GSK3β (Ser9, CST, Danvers, MA, United States) activity in accordance with the manufacturers’ guidelines.

### *In vitro* BACE-1 Assay

The ability of LC1405 at concentrations ranging from 0.3 and 300.0 μM to inhibit beta-site APP-cleaving enzyme (BACE-1) activity was determined using a BACE-1 assay kit according to the manufacturer’s guidelines.

### Statistical Analysis

Data were analyzed using SPSS software (version 18.0, SPSS, Inc., Chicago, IL, United States), and presented as mean ± standard error of the mean (SEM). Statistical analysis was performed by using different tests as follows: (1) escape latency of the MWM test the acquisition trial was analyzed using the ANOVA for repeated measures (training days and treatment, with treatment as main effect), and one-way ANOVA with Tukey’s *post hoc* analyses were used to analyze treatment differences (treatment as main effect); (2) treatment differences in the probe trials, pathological and biochemical assays, and *in vitro* studies were performed using a one-way ANOVA, followed by Tukey’s *post hoc* testing to analyze the differences between groups; (3) the area under the curve (AUC) of histamine and ACh levels in the brain after LC1405 administration were analyzed by student *t*-test. The statistical significance was set at a *p*-value of less than 0.05.

## Results

### LC1405 Treatment Improves Cognitive Deficits in APP/PS1 Mice

At 13 months of age, APP/PS1 mice underwent behavioral testing using the MWM test, a widely-accepted test for spatial learning and memory capability. During the acquisition trial, the escape latency to the target platform for all mice is illustrated in Figure 1B. Significant differences on escape latency within groups [*F*_(4,192)_ = 138.697, *p* < 0.001] and a significant treatment effect on escape latency were found [*F*_(3,48)_ = 20.572, *p* < 0.001]. Subsequent *post hoc* comparison illustrated that treatment with 3 mg/kg of LC1405 was considerable in improving the spatial learning ability in APP/PS1 mice compared to APP/PS1 control mice (*p* < 0.05). Probe trials were carried out to evaluate short-term memory at 2 h and long-term memory at 48 h following the five-consecutive-day trials. Our data indicated that APP/PS1 control mice stayed for a shorter time and completed fewer crossings in the target quadrant at both time points compared with WT control mice (Figure 1C,D) (2 h: 18.77 ± 1.25% vs. 30.14 ± 3.04%, 1.38 ± 0.31 vs. 3.46 ± 0.52, *p* < 0.001 and 0.01; 48 h: 17.94 ± 0.85% vs. 32.51 ± 1.37%, 1.38 ± 0.29 vs. 3.62 ± 0.24, both *p* < 0.001), while LC1405-treated APP/PS1 mice stayed in the target quadrant for a longer time (2 h: 27.29 ± 1.47% vs. 18.77 ± 1.25%, *p* < 0.01; 48 h: 26.51 ± 0.87% vs. 17.94 ± 0.85%, *p* < 0.001), and performed more crossings at the platform location compared with APP/PS1 control mice (2 h: 3.00 ± 0.29 vs. 1.38 ± 0.31, *p* < 0.05; 48 h: 2.92 ± 0.28 vs. 1.38 ± 0.29, *p* < 0.01). Moreover, our results demonstrated no significant differences in swimming speed among treatment groups in the acquisition trial (Figure 1E), suggesting that the improvement on learning capability in LC1405-treated APP/PS1 mice was not due to locomotor ability.

During the retention trial in the passive avoidance test, APP/PS1 control mice re-entered the illuminated compartment more frequently with shorter step-through latency compared to WT control mice (Figure 1F,G) (151.58 ± 24.22 s vs. 236.57 ± 24.14 s, 3.22 ± 0.82 vs. 1.11 ± 0.45, both *p* < 0.05,), while LC1405-treated APP/PS1 mice showed less tendency toward the illuminated compartment, involving longer step-through latency and fewer numbers of errors compared to APP/PS1 control mice (220.75 ± 26.25 s vs. 151.58 ± 24.22 s, 1.44 ± 0.58 vs. 3.22 ± 0.82, both *p* < 0.05), indicating that LC1405 treatment ameliorated memory dysfunction.

In WT mice, LC1405 treatment did not affect cognitive capabilities in the behavioral tests performed, therefore we concluded that long-term oral administration of LC1405 plays a role in ameliorating Aβ-dependent cognitive deficits rather than affecting functions that are normal.

### LC1405 Prevents Neuronal Degeneration and Protects Ultrastructure, but Has No Substantial Effect on the Reduction of Aβ Burden or Oxidative Markers in APP/PS1 Mice

**FIGURE 2:**
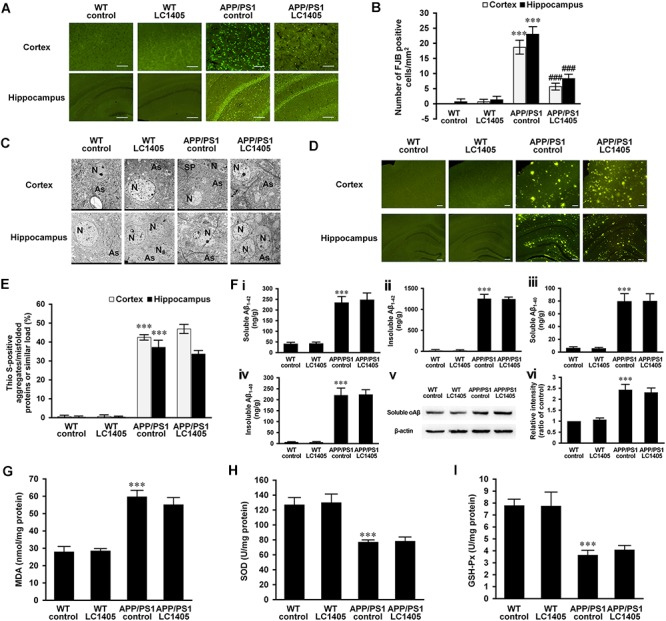
LC1405 prevents neurodegeneration, protects neuronal ultrastructure, but has no effect on Aβ burden and oxidative stress in APP/PS1 mice. **(A)** Representative images of Fluoro-Jade B (FJB) staining of neurodegeneration in the cerebral cortex and hippocampus. Scale Bar = 100 μM. **(B)** Mean number of FJB-positive cells/mm^2^ in the cortex and hippocampus (*n* = 3). **(C)** Representative images of the neuropil ultrastructure in the cortex and hippocampus of WT and APP/PS1 mice. As, astrocytes; N, neuron; SP, senile plaques. **(D)** Representative images of thioflavin S (Thio-S) staining. Scale Bar = 100 μM. **(E)** Quantitative analysis of positive Thio-S staining of aggregates/misfolded proteins or similar (*n* = 3). **(F)** Soluble and insoluble Aβ_1-42_ **(i, ii)** and _1-40_ **(iii, iv)** levels in brain homogenates by ELISA, and soluble oAβ **(v, vi)** level in brain homogenates by Western blot (*n* = 4). **(G–I)** Measurement of oxidative markers MDA, SOD, and GSH-Px in brain homogenates (*n* = 4). Data are presented as mean ± SEM. ^∗∗∗^*p* < 0.001 vs. WT control,^###^*p* < 0.001 vs. APP/PS1 control.

FJB is a useful marker for the identification of neuronal degeneration because of its ability to stain the entire neuron, such as the cell body, dendrites, axons, and axon terminals (Ballok et al., 2003). In both the cerebral cortex and the hippocampus of WT control and LC1405-treated WT mice, FJB-positive neurons were rarely detected. However, in the cortical and hippocampal regions of APP/PS1 mice, the number of FJB-positive neurons was significantly increased (Figure 2A,B) (cortex: 18.67 ± 2.02 vs. 0; hippocampus: 23.00 ± 2.30 vs. 0.67 ± 0.67; both *p* < 0.001**)** compared to WT control mice. LC1405 treatment decreased the number of FJB-positive neurons in the cortex and hippocampus of APP/PS1 mouse brains compared to APP/PS1 control group (cortex: 5.67 ± 0.88 vs. 18.67 ± 2.02; hippocampus: 8.33 ± 1.20 vs. 23.00 ± 2.30; both *p* < 0.001), indicating that LC1405 treatment relieved the degenerative pathology in APP/PS1 mouse brain.

To further evaluate degenerative changes at the ultrastructural level, sections of the cortex and hippocampus were observed using a transmission electron microscope (Figure 2C). The cortical and hippocampal regions of WT control and LC1405-treated WT mice were well-characterized, showing a compact neuropil appearance with normal neurons (N) and astrocytes (As), with no swelling or shrinkage. In APP/PS1 mouse brain regions, neuropils were disrupted and neuronal degeneration was detected as the neurons showed a shrunken appearance. Degenerative neurons were embodied in the ruptured neuronal membranes, with cytoplasm containing dark granules, and condensed nuclei. In addition to degeneration of adjacent neuropils, intracellular vacuolation and astrocyte edema were observed, accompanied with Aβ plaques (SP) deposited in the neuropils nearby. LC1405 treatment attenuated neuropil degeneration in the cortex and hippocampus of LC1405-treated APP/PS1 mice. Signs of swelling and disintegration of astrocytes were disappeared, and Aβ plaque deposition was less apparent in surrounding neuropils. Together, these findings indicated that LC1405 treatment was effective in preventing Aβ-mediated neuronal degeneration.

Overproduction and aggregation of Aβ are considered key pathological events in the neurodegenerative cascade in AD. Although in this study neuronal degeneration and neuropil ultrastructure were ameliorated by LC1405 treatment, Aβ aggregates/misfolded proteins or similar in the cortex and hippocampus as indicated by Thio S staining, were not reduced after long-term oral administration of LC1405 (Figure 2D,E) (cortex: 46.93 ± 1.98 vs. 42.27 ± 1.71; hippocampus: 33.67 ± 1.43 vs. 37.23 ± 3.32). Similarly, the levels of soluble or insoluble Aβ_1-42_ or Aβ_1-40_ in the brain did not significantly decrease following LC1405 treatment (Figure 2Fi–iv) (soluble Aβ_1-42_: 246.80 ± 28.48 ng/g vs. 233.47 ± 25.05 ng/g; insoluble Aβ_1-42_: 1236.65 ± 34.57 ng/g vs. 1246.66 ± 87.17 ng/g; soluble Aβ_1-40_: 79.57 ± 10.45 ng/g vs. 79.18 ± 11.08 ng/g; insoluble Aβ_1-40_: 221.98 ± 20.29 ng/g vs. 218.87 ± 31.29 ng/g). Furthermore, the increased level of A11-immunoreactive prefibrillar oligomers, which represented the presence of potential neurotoxic Aβ oligomers and then underwent a concerted conformation change from protofibrils to form fibrils (Glabe, 2008; Gulisano et al., 2018), could not be significantly decreased by LC1405 treatment either (Figure 2Fv,vi) (ratio of WT control: 2.31 ± 0.20 vs. 2.43 ± 0.25). Combined, these data suggested that LC1405 treatment did not have a substantial effect in reducing Aβ levels in APP/PS1 mouse brain.

Oxidative stress is implicated in the pathology of AD, and in this study, the APP/PS1 mouse model displayed alterations in markers of oxidative stress in the brain, such as MDA, SOD, and GSH-Px compared to WT control group (Figure 2G–I) (MDA: 59.63 ± 3.32 nmol/mg protein vs. 27.87 ± 2.69 nmol/mg protein; SOD: 76.33 ± 2.25 U/mg protein vs. 126.78 ± 8.72 U/mg protein; GSH-Px: 3.62 ± 0.35 U/mg protein vs. 7.78 ± 0.47 U/mg protein; all *p* < 0.001). However, these markers were not significantly ameliorated in APP/PS1 mouse brain after treatment with LC1405 (MDA: 55.03 ± 3.71 nmol/mg protein vs. 59.63 ± 3.32 nmol/mg protein; SOD: 78.72 ± 6.10 U/mg protein vs. 76.33 ± 2.25 U/mg protein; GSH-Px: 4.06 ± 0.31 U/mg protein vs. 3.62 ± 0.35 U/mg protein).

### LC1405 Treatment Does Not Alter AChE Activity in APP/PS1 Mouse Brain Tissue, but Increases the Level of ACh and Improves the Release of Histamine and ACh

**FIGURE 3:**
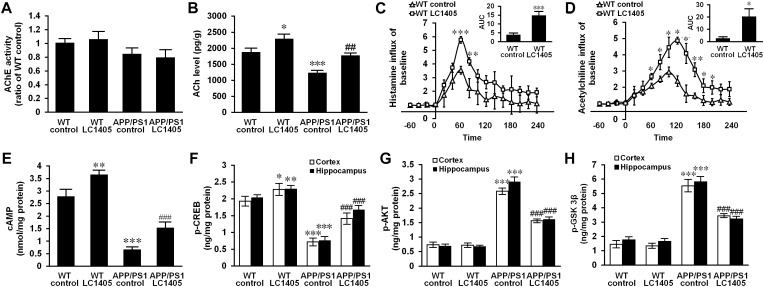
LC1405 treatment increases the level of ACh and histamine and modifies H3R-mediated signaling in APP/PS1 mouse brain. **(A)** LC1405 treatment did not alter AChE activity in mouse brain (*n* = 4). **(B)** LC1405 treatment increased ACh level in the brain of WT and APP/PS1 mice (*n* = 4). **(C,D)** LC1405 treatment stimulated histamine and ACh responses in WT mouse brain (*n* = 4). **(E)** LC1405 treatment increased cAMP levels in WT and in APP/PS1 mouse brain (*n* = 4). **(F)** LC1405 treatment up-regulated p-CREB level in the cerebral cortex and hippocampus of WT and APP/PS1 mice (*n* = 4). **(G,H)** LC1405 treatment increased the signaling transduction of AKT/GSK3β in the cerebral cortex and hippocampus of APP/PS1 mice (*n* = 4). Data are presented as mean ± SEM. ^∗^*p* < 0.05, ^∗∗^*p* < 0.01, ^∗∗∗^*p* < 0.001 vs. WT control, ^##^*p* < 0.01, ^###^*p* < 0.001 vs. APP/PS1 control.

Due to an early and severe depletion of cholinergic innervations in AD pathology, a decrease in AChE activity in the brain is a consistent finding (Dumas and Newhouse, 2011; Carvajal et al., 2013). A slight decrease in AChE activity was observed in APP/PS1 mouse brain, and LC1405 treatment did not alter the AChE activity significantly either in APP/PS1 or in WT mouse brain (Figure 3A) (LC1405-treated WT vs. WT control: 1.05 ± 0.10 vs. 1.00 ± 0.06; LC1405-treated APP/PS1 vs. APP/PS1 control: 0.79 ± 0.11 vs. 0.84 ± 0.08). Up-regulated ACh levels were observed in LC1405-treated WT and APP/PS1 mice compared to the correspondent LC1405-untreated groups (Figure 3B) (LC1405-treated WT vs. WT control: 2285.69 ± 123.96 pg/g vs. 1900.43 ± 82.70 pg/g, *p* < 0.05; LC1405-treated APP/PS1 vs. APP/PS1 control: 1761.88 ± 59.18 pg/g vs. 1222.23 ± 62.42 pg/g, *p* < 0.01), suggesting that LC1405 might be effective in increasing the responsiveness of ACh in cholinergic neurons.

In subsequent studies, the concentration of histamine and ACh were compared in the brain of WT mice with and without LC1405 treatment. After LC1405 administration, changes in ACh and histamine levels were expressed in terms of the area under the curve (AUC) for a 0 to 4 h time-period. The time course results of histamine following LC1405 treatment revealed its rapid release that peaked within 30–60 min of dosing and persisted over the 4 h test period. In addition, ACh levels increased over the 60-min period after administration. When comparing the responses with the results of WT control mice, significant increases in AUC in histamine and ACh levels were observed after administration of 3 mg/kg LC1405 (Figure 3C,D) (histamine: 14.61 ± 2.05 vs. 3.72 ± 0.72, *p* < 0.001; ACh: 20.27 ± 5.64 vs. 2.64 ± 0.80, *p* < 0.05). This was not only the maximal response of ACh after LC1405 treatment, but a sustained increase in the brain after treatment from 60 to 120 min. The response of ACh could be a consequence of increased histamine release. Therefore, these results suggested that LC1405 might act on H3R, thereby leading to an overall increase in histamine and ACh levels.

### LC1405 Treatment Modifies H3R-Mediated Signaling in APP/PS1 Mouse Brain

To identify whether H3R inhibition was a result of the treatment with LC1405, the downstream signaling pathways of H3R coupling were evaluated to assess the potential therapeutic efficacy. Our results showed that transduction of the cAMP/CREB pathway changed significantly in the brains of APP/PS1 mice, which showed a significant decrease in cAMP and p-CREB levels, of 76.12 and 62.78%, respectively in 13-month-old APP/PS1 mice when compared with the WT control (Figure 3E,F, all *p* < 0.001). The Tukey’s *post hoc* comparison demonstrated that oral administration of LC1405 resulted in increased cAMP levels and upregulated p-CREB activity in APP/PS1 mouse brain compared to APP/PS1 control group (cAMP: 1.55 ± 0.17 nmol/mg protein vs. 0.66 ± 0.06 nmol/mg protein; p-CREB: 1.41 ± 0.14 ng/mg protein vs. 0.72 ± 0.09 ng/mg protein, 1.68 ± 0.11 ng/mg protein vs. 0.76 ± 0.09 ng/mg protein; all *p* < 0.001). It should be noted that LC1405 treatment also activated cAMP/CREB cascades in WT control mice, indicating that an oral dose of 3 mg/kg of LC1405 affected basal transduction in normal brain (cAMP: 3.66 ± 0.13 nmol/mg protein vs. 2.79 ± 0.25 nmol/mg protein; p-CREB: 2.28 ± 0.15 ng/mg protein vs. 1.93 ± 0.12 ng/mg protein, 2.29 ± 0.08 ng/mg protein vs. 2.04 ± 0.07 ng/mg protein; *p* < 0.05-0.01). Thus, cAMP/CREB pathway transduction following H3R inhibition might play a role in the action of LC1405.

To investigate H3R downstream signaling, modification of the AKT/GSK3β axis was evaluated. Up-regulation of phosphorylated AKT was accompanied by a high level of GSK3β phosphorylation in the cortex and hippocampus of APP/PS1 mice compared to WT control group (Figure 3G,H) (p-AKT: 2.58 ± 0.09 ng/mg protein vs. 0.73 ± 0.06 ng/mg protein, 2.91 ± 0.15 ng/mg protein vs. 0.69 ± 0.04 ng/mg protein; p-GSK3β: 5.54 ± 0.40 ng/mg protein vs. 1.45 ± 0.20 ng/mg protein, 5.82 ± 0.31 ng/mg protein vs. 1.76 ± 0.15 ng/mg protein; all *p* < 0.001). However, LC1405 treatment suppressed the phosphorylation in APP/PS1 mice (p-AKT: 1.56 ± 0.04 ng/mg protein vs. 2.58 ± 0.09 ng/mg protein, 1.61 ± 0.05 ng/mg protein vs. 2.91 ± 0.15 ng/mg protein; p-GSK3β: 3.44 ± 0.09 ng/mg protein vs. 5.54 ± 0.40 ng/mg protein, 3.22 ± 0.11 ng/mg protein vs. 5.82 ± 0.31 ng/mg protein; all *p* < 0.001), suggesting that LC1405 might be able to reverse the activated signaling of AKT/GSK3β in AD.

### LC1405 Protects Neuronal Cells Against Copper-Induced Aβ Toxicity *in vitro*

**FIGURE 4:**
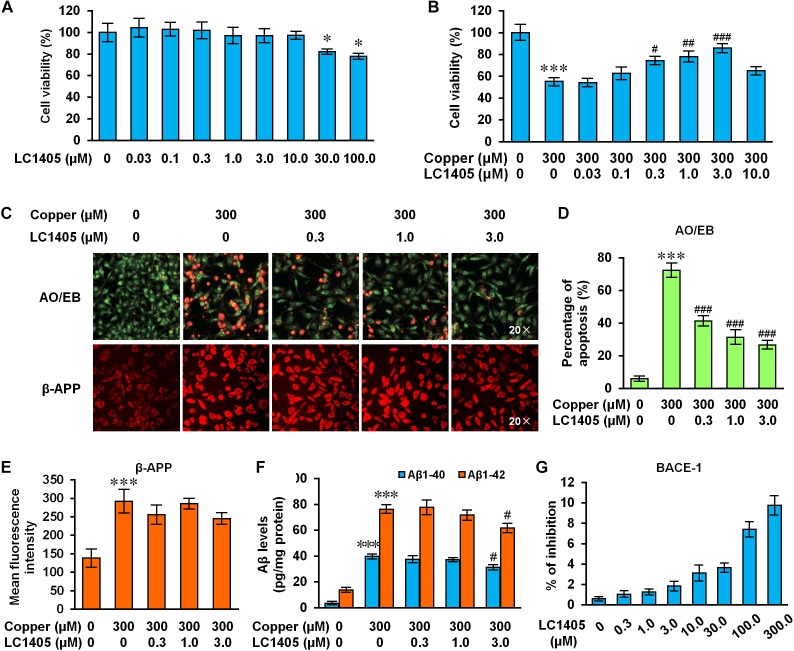
LC1405 protects APPsw cells against copper-induced Aβ toxicity. **(A)** Non-toxic concentrations of LC1405 that could be safely used to treat APPsw cells for 24 h without copper treatment (*n* = 8). **(B)** Treatment with LC1405 increased cell viability as evaluated by MTS cell proliferation assay (*n* = 8). **(C)** Representative images of acridine orange (AO)/ethidium bromide (EB) and β-APP staining (×20 magnification). **(D)** LC1405 treatment decreased the percentage of apoptotic cells in copper-treated APPsw cells (*n* = 4). **(E)** LC1405 treatment affected the mean fluorescence intensity of β-APP in copper-treated APPsw cells (*n* = 4). **(F)** LC1405 treatment inhibited the content of Aβ_1-40/42_ in copper-treated APPsw cells (*n* = 4). **(G)** Higher concentrations of LC1405 resulted in slight BACE-1 inhibition. Data are expressed as mean ± SEM. ^∗^*p* < 0.05, ^∗∗∗^*p* < 0.001 vs. control cells, ^#^*p* < 0.05,^##^*p* < 0.01, ^###^*p* < 0.001 vs. copper-treated cells.

To better mimic Alzheimer’s deficits, copper treatment was used to study metal ion imbalance triggering Aβ neurotoxicity using cells overexpressing the Swedish mutant form of human APP (Zhang et al., 2006; Liu et al., 2012). Before evaluating the protective effects of LC1405, we first established its safe, non-toxic dose in APPsw cells without copper treatment. Our results indicated that LC1405 concentration ranging between 0.03 and 10.0 μM over 24 h did not have any toxic effect (Figure 4A). However, in the presence of 300 μM copper, LC1405 significantly increased cell viability at 0.3 μM, 1.0 μM, and 3.0 μM to in a concentration-dependent manner (Figure 4B) (74.46 ± 2.99%, 78.15 ± 4.12%, 85.93 ± 3.40% vs. 55.30 ± 3.22%, *p* < 0.05-0.001).

Similar findings were observed in cells stained with the AO/EB apoptosis-detection dye. The normal morphology of control cells was characterized by the presence of green-colored nuclei and an intact structure, whereas apoptotic characteristics included shrinkage, membrane blebbing, chromatin condensation, and the formation of apoptotic bodies were found in copper-treated APPsw cells. In the presence of copper, the proportion of APPsw cells undergoing apoptosis was 72.43 ± 3.76% compared to control cells (Figure 4C,D, *p* < 0.001). LC1405 treatment at concentrations of 0.3 μM, 1.0 μM, and 3.0 μM rescued the morphological changes indicative of apoptosis, and reduced the percentage of cells positively stained for the apoptosis-detection dye in a concentration-dependent manner compared with the cells treated with copper (41.41 ± 2.55%, 31.42 ± 3.78%, 26.77 ± 1.92% vs. 72.43 ± 3.76%, all *p* < 0.001). Combined with these protective effects, LC1405 concentrations at 0.3 μM, 1.0 μM, and 3.0 μM were chosen to investigate the underlying mechanism of LC1405 protection in the AD *in vitro* model.

### LC1405 Has Limited Effects on Decreasing β-APP Expression or Attenuating Aβ_1-40/42_ Levels

Aβ peptides are primarily generated from the cleavage of APP, which represent the major pathological event in the development of AD. In the present study, administration of copper increased the expression of β-APP by 2.11 fold. Similarly, the levels of Aβ_1-40_ and Aβ_1-42_ in APPsw cells increased respectively by 11.65 and 5.56 fold (Figure 4C,E,F, all *p* < 0.001). Treatment with LC1405 at the tested concentrations did not significantly reduce the expression of β-APP or the levels of Aβ_1-40_ and Aβ_1-42_ in APPsw cells, except at the highest concentration of 3.0 μM that showed an effect on inhibiting Aβ_1-40/42_ levels by the reduction of 21.35% and 23.15% (Figure 4F, both *p* < 0.05). Moreover, LC1405 at higher concentrations (100 and 300 μM) resulted in a slight BACE-1 inhibition (Figure 4G, 7.42 ± 0.64% and 9.76 ± 0.83%), what was outside the range of concentrations tested in AD cells. Thus, our results suggested that LC1405 treatment might be independent of Aβ overproduction in the β-amyloidogenic pathway.

### H3R Inhibition Contributes to the Neuroprotective Effects of LC1405 Against Aβ-Induced Toxicity

LC1405 might be identified as a candidate H3R antagonist with neuroprotection through virtual screening, serial cell-based assays, and extensive neuroprotection evaluation. In view of the Bayesian prediction, LC1405, as a potential H3R ligand, serves as a reference compound (Supplementary Table 2). In target functional activity assays *in vitro*, LC1405 showed substantial antagonistic effects combined with a relative high affinity for human and rat H3R, but relatively lower affinities for H1, H2, and H4 subtypes (Supplementary Table 2). Furthermore, LC1405 itself blocked the decrease of cAMP induced by RAMH (Supplementary Table 2), indicating that LC1405 might interact with H3R, thereby directly increasing cAMP levels. Importantly, LC1405 treatment exerted neuroprotective effects on rat primary cortical neurons against Aβ_25-35_- and fibrillar Aβ_1-40_ (fAβ_1-40_)-induced toxicity at 0.3 μM, 1.0 μM, and 3.0 μM in a concentration-dependent manner (Supplementary Figure 2, *p* < 0.05-0.001).

**FIGURE 5:**
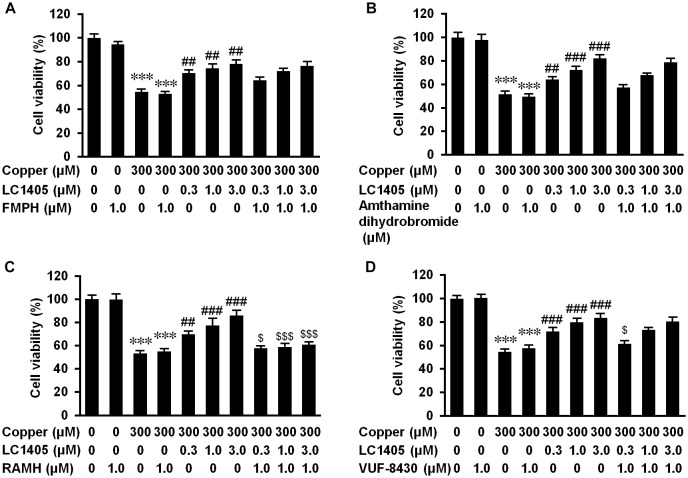
H3R contributes to LC1405 neuroprotection against copper-mediated Aβ toxicity. Histamine receptors were pharmacologically activated by their specific agonists. The specific histamine receptor agonists activating: **(A)** H1, **(B)** H2 and **(D)** H4 subtypes did not clearly block the increased cell viability due to LC1405 after exposure to copper, whereas activation of **(C)** H3R with RAMH blocked the increased cell viability at all tested concentrations of LC1405. Data are expressed as mean ± SEM. *n* = 8. ^∗∗∗^*p* < 0.001 vs. control cells, ^##^*p* < 0.01, ^###^*p* < 0.001 vs. copper-treated cells, ^$^*p* < 0.05, ^$$$^*p* < 0.001 vs. copper+LC1405 treated cells.

To determine whether H3R contributed to LC1405-mediated neuroprotection against Aβ-induced cytotoxicity, specific H3R agonist and other histamine receptor subtype agonists were used. As shown in Figure 5C, pharmacological activation of H3R with RAMH blocked the ability of LC1405 to rescue the decreased cell viability observed after exposure to copper at all the tested concentrations (57.84 ± 1.07% vs. 69.80 ± 1.56%, 58.96 ± 1.63% vs. 77.44 ± 5.07%, 60.91 ± 1.15% vs. 86.01 ± 3.27%, *p* < 0.05-0.001). Other specific histamine receptor agonists that activated H1, H2, and H4 subtypes did not exert any substantial effect (Figure 5A,B) or only partly reduced cell viability that had been increased by LC1405 (Figure 5D, 61.40 ± 1.66% vs. 71.87 ± 2.13%, *p* < 0.05: copper-injured APPsw cells treated with LC1405 and VUF-8430 vs. copper-injured APPsw cells treated with LC1405). Thus, these results suggested that neuroprotection due to LC1405 against copper-induced Aβ injury might be largely attributable to its inhibitory effects on H3R.

### H3R Mediates the Neuroprotective Effect of LC1405 Against Aβ Injury Through cAMP/CREB and PI3K/AKT/GSK3β Signaling Pathways

**FIGURE 6:**
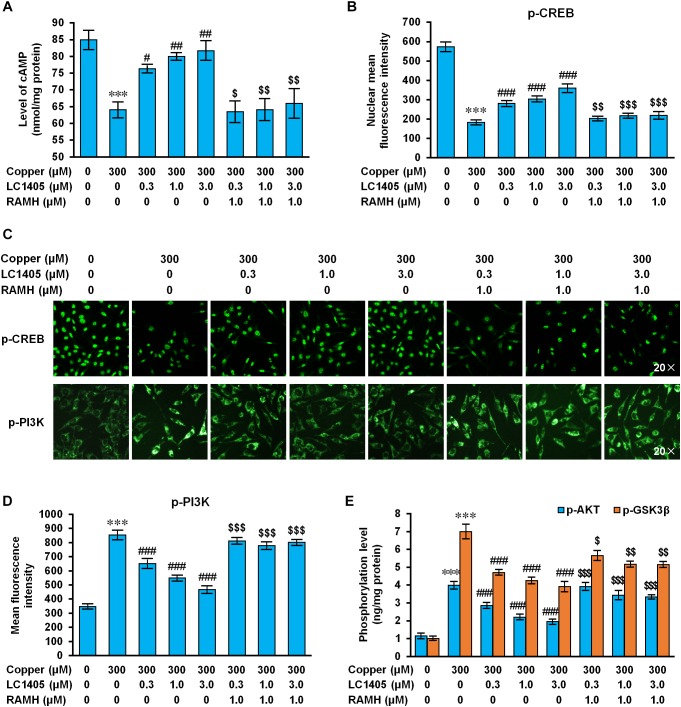
LC1405 regulates H3R-mediated signaling pathways against Aβ-induced toxicity. **(A)** LC1405 treatment did not increase the cAMP level in APPsw cells pre-treated with RAMH (*n* = 4). **(B)** LC1405 treatment did not increase the mean fluorescence intensity of p-CREB in APPsw cells against copper-induced Aβ toxicity pre-treated with RAMH (*n* = 4). **(C)** Representative immunohistochemical images of p-CREB and p-PI3K staining ( × 20 magnification). **(D)** LC1405 treatment of copper-induced Aβ toxicity of APPsw cells pre-treated with RAMH did not decrease the mean fluorescence intensity of p-PI3K (*n* = 4). **(E)** Effects of increasing phosphorylation of AKT and GSK3β by LC1405 were inhibited by pre-treatment with RAMH (*n* = 6). Data are expressed as mean ± SEM. ^∗∗∗^*p* < 0.001 vs. control cells, ^#^*p* < 0.05,^##^*p* < 0.01, ^###^*p* < 0.001 vs. copper-treated cells. ^$^*p* < 0.05,^$$^*p* < 0.01, ^$$$^*p* < 0.001 vs. copper+LC1405 treated cells.

Histamine H3 receptor signaling caused negative coupling of AC, thereby decreasing the intracellular activity of the cAMP/PKA pathway, subsequently reducing CREB levels (Bhowmik et al., 2014). In APPsw cells subjected to copper, a significant reduction in the intracellular of cAMP level and a significant decrease in phosphorylated CREB level were observed compared to the ones without copper treatment (Figure 6A–C) (cAMP: 64.04 ± 2.06 nmol/mg protein vs. 84.92 ± 2.51 nmol/mg protein; p-CREB: 182.82 ± 7.39 vs. 772.98 ± 19.03; both *p* < 0.001), indicating that the cAMP/CREB signaling pathway was inhibited in response to copper-triggered Aβ toxicity. LC1405 treatment contrasted Aβ-neurotoxicity by increasing intracellular levels of cAMP and up-regulating phosphorylated CREB, both in a concentration-dependent manner (cAMP: 76.27 ± 0.91 nmol/mg protein, 79.97 ± 0.84 nmol/mg protein, 81.71 ± 2.61 nmol/mg protein vs. 64.04 ± 2.06 nmol/mg protein; p-CREB: 280.07 ± 9.85, 302.98 ± 11.49, 359.74 ± 16.54 vs. 182.82 ± 7.39; *p* < 0.05-0.001). However, these effects of LC1405 were abolished at all the tested concentrations by the pharmacological activation of H3R treated with RAMH (cAMP: 63.48 ± 2.88 nmol/mg protein vs. 76.27 ± 0.91 nmol/mg protein, 64.10 ± 2.95 nmol/mg protein vs. 79.97 ± 0.84 nmol/mg protein, 65.92 ± 4.11 nmol/mg protein vs. 81.71 ± 2.61 nmol/mg protein; p-CREB: 202.51 ± 6.27 vs. 280.07 ± 9.85, 217.85 ± 7.47 vs. 302.98 ± 11.49, 218.85 ± 14.51 vs. 359.74 ± 16.54; *p* < 0.05-0.001), suggesting the participation of the cAMP/CREB pathway following H3R signaling in the reduction of Aβ-mediated deficits by LC1405 using copper to trigger neurotoxicity of Aβ.

Histamine H3 receptor is related to PI3K/AKT/GSK3β cascade that play a role in neuronal survival, thereby exerting neuroprotection on lesions due to multiple cytotoxic factors (Provensi et al., 2016). Copper-triggered Aβ exposure increased the phosphorylated level of PI3K, AKT, and GSK3β (Figure 6C–E) (p-PI3K: 853.62 ± 26.34 vs. 347.84 ± 10.75; p-AKT: 3.98 ± 0.16 ng/mg protein vs. 1.23 ± 0.11 ng/mg protein; p-GSK3β: 7.00 ± 0.36 ng/mg protein vs. 1.00 ± 0.07 ng/mg protein; all *p* < 0.001), whereas LC1405 treatment at the concentration of 0.3 μM, 1.0 μM, and 3.0 μM prevented the increased phosphorylation of PI3K, AKT, and GSK3β in APPsw cells after exposure to copper (p-PI3K: 651.74 ± 26.29, 548.64 ± 12.29, 467.27 ± 18.17 vs. 853.62 ± 26.34; p-AKT: 2.85 ± 0.12 ng/mg protein, 2.20 ± 0.10 ng/mg protein, 1.94 ± 0.08 ng/mg protein vs. 3.98 ± 0.16 ng/mg protein; p-GSK3β: 4.70 ± 0.11 ng/mg protein, 4.25 ± 0.13 ng/mg protein, 3.90 ± 0.23 ng/mg protein vs. 7.00 ± 0.36 ng/mg protein; all *p* < 0.001). However, in response to RAMH, H3R activation affected the LC1405 effect of preventing the increased phosphorylation of PI3K, AKT, and GSK3β induced by copper (p-PI3K: 810.88 ± 16.18 vs. 651.74 ± 26.29, 778.13 ± 19.69 vs. 548.64 ± 12.29, 801.26 ± 13.68 vs. 467.27 ± 18.17; p-AKT: 3.92 ± 0.15 ng/mg protein vs. 2.85 ± 0.12 ng/mg protein, 3.42 ± 0.21 ng/mg protein vs. 2.20 ± 0.10 ng/mg protein, 3.33 ± 0.06 ng/mg protein vs. 1.94 ± 0.08 ng/mg protein; p-GSK3β: 5.66 ± 0.22 ng/mg protein vs. 4.70 ± 0.11 ng/mg protein, 5.17 ± 0.11 ng/mg protein vs. 4.25 ± 0.13 ng/mg protein, 5.15 ± 0.12 ng/mg protein vs. 3.90 ± 0.23 ng/mg protein; *p* < 0.05-0.001). These observations indicated that H3R-mediated PI3K/AKT/GSK3β signaling is involved in the protective role of LC1405 against copper-induced Aβ cytotoxicity.

Based on the observations above, LC1405 protected neurons against copper-triggered Aβ-induced toxicity through H3R-dependent cAMP/CREB and PI3K/AKT/GSK3β signaling.

## Discussion

Two major contributions are provided by this study to elucidate the underlying mechanism of action of LC1405. First, LC1405 was identified as a prospective H3R antagonist that prevented Aβ-induced neurotoxicity and as a potential treatment of AD both *in vitro* and *in vivo*. Second, a cellular signaling profile of LC1405 in H3R antagonism was provided, characterized by a beneficial H3R-dependent signaling of cAMP/CREB and AKT/GSK3β axes. These findings revealed novel evidence and insights that focus on the role of H3R, which might be a potential therapeutic target in the treatment of AD.

From a previous multi-step screening process, LC1405 was identified as a potential H3R-targeting compound as a result of *in silico* prediction of H3R ligands, and subsequent cell-based-target assays on H3Rs and *in vitro* neuroprotective evaluation (see Supplementary Material). Furthermore, the ability of LC1405 in reversing the effects of AD and underlying mechanisms were evaluated in APP/PS1 double transgenic mice, and SH-SY5Y cells that express APP with a familial Swedish mutation, both excessively expressing the APP gene and mimicking β-amyloidogenic disturbance (Zhang et al., 2006; Voss et al., 2014).

Our study found that LC1405 improved learning and memory deficits in AD mice (Figure 1B–G). Consistent with these findings, neurodegeneration and ultrastructural abnormalities were rescued in the hippocampal and cortical regions (Figure 2A–C). Importantly, these alterations were in line with the *in vitro* cytoprotection and apoptotic preservation demonstrated after LC1405 treatment (Figure 4A–D). Thus, treatment with LC1405 is a promising approach in ameliorating the pathology of AD.

Excessive oxidation has been recognized as a contributor to Aβ-induced neurotoxicity with metal dyshomeostasis being implicated in the Aβ aggregation process of AD. Extremely high ion concentrations, such as copper, have been found colocalized with Aβ deposits in the AD-affected brain (Jakob-Roetne and Jacobsen, 2009; Duce and Bush, 2010). In addition, redox active copper ions lead to ROS overproduction, resulting in oxidative damage that triggers neurodegeneration of the brain (Huang et al., 1999; Jakob-Roetne and Jacobsen, 2009). Here, limited antioxidative effects were obtained after LC1405 treatment in APP/PS1 mice, as indicated by the limited reduction in oxidative biomarkers, such as MDA, SOD, and GSH-Px (Figure 2G–I). Thus, LC1405 did not provide a sufficient antioxidant effect through scavenging ROS generation due to Aβ-mediated neurotoxicity related with AD.

The widely accepted amyloid hypothesis suggests that the assembly of Aβ to form aggregates, involving oligomers, protofibrils, fibrils, and even senile plaques, is a central event in the progression of AD (Ferreira et al., 2015; Gulisano et al., 2018). Various reports also confirmed that aggregated Aβ are toxic to neuronal cells, thereby indicating that inhibition of Aβ aggregation as a potential AD therapy is a reasonable strategy in AD treatment. However, the results from pathological, biological, and western blotting experiments showed that LC1405 treatment did not provide a significant reduction in Aβ burden in APP/PS1 mice (Figure 2C–F), which resulted in Aβ plaque deposition as early as 2 months of age and moderate levels of Aβ deposition at the age of 5 months (Blanchard et al., 2003; Ding et al., 2008).

Apart from Aβ aggregation and deposition, the β-amyloidogenic pathway in which Aβ is produced through APP cleavage by BACE1 also plays a role in the evaluation of neuroprotective agents. LC1405 was found to possess a weak inactivation effect on BACE-1 (Figure 4G). This result partly explained the limited down-regulation of APP expression *in vitro* and failure of lowering multiple forms of Aβ levels, including soluble and insoluble Aβ, A11-immunoreactive prefibrillar oligomers, and amyloid aggregates/misfolded proteins or similar (Figure 2D–F, 4C,E,F). Therefore, we initially presumed that the beneficial effect of LC1405 on enhancing learning capacity and reducing memory deficits might be independent of changes in the amyloidogenic processing of APP, or in the process of secretion or deposition of Aβ. Although we found that LC1405 was effective in recuing neuronal cell viability due to fibrillar Aβ_1-40/42_ toxicity (Supplementary Figure 2), whether LC1405 had the definite effect on altering aggregation profile of Aβ should be further studied on the synthetic peptide and Alzheimer-related animal models.

In the part of the brain that is responsible for learning and memory, H3R, both for integrity and function, is conserved in Aβ over-expressing regions (Foley et al., 2009), indicating that H3R may neither participate in the amyloidogenic processing of APP nor alter the secretion or deposition of Aβ. In our study, LC1405 might preferentially produce improvements in cognition and neuroprotective effects by altering H3R-dependent processes rather than by reducing Aβ deposition or decreasing oxidative stress. Two potential beneficial effects of LC1405 involved in preventing Aβ toxicity were identified in this work: LC1405 (i) improved cholinergic activity by up-regulating ACh and histamine release; (ii) maintained cognitive molecular cascades of H3R-dependent transduction, involving cAMP/CREB and AKT/GSK3β signaling pathways.

Histamine H3 receptor is an auto- and heteroreceptor that negatively regulates the release of histamine and several cognition-related key neurotransmitters, such as ACh, which is recognized as a major neurochemical modulator of cognitive processing, particularly in AD (Bartus, 2000). In this study, we found that oral administration of LC1405 increased the levels of histamine and ACh in mouse brain (Figure 3C,D), which was in accordance with the hypothesis that H3R antagonists increased neurotransmitter levels involving ACh and histamine in the brain, thus counteracting AD deficits (Bitner et al., 2011). H3Rs on histaminergic neurons provide tonic inhibition of the firing rate (Jin and Panula, 2005), whereas those on presynaptic histaminergic terminals restrict histamine synthesis and release (Arrang et al., 1983, 1987). Therefore, our hypothesis was that LC1405 administration might inhibit presynaptic H3 autoreceptor activation and increase histamine levels in the synaptic cleft. In addition, long-term administration of LC1405 increased ACh levels in the brain without the influence of AChE activity (Figure 3A,B). Since an *in vivo* micro-dialysis assay suggested that hetero-H3R-mediated regulation of ACh is related to potassium levels in different regions of the brain (Blandina et al., 1996), but not by counteracting the activation of histamine H1 or the H2 subtype (Esbenshade et al., 2008), our conclusion was that in response to Aβ toxicity, LC1405 treatment might increase endogenous ACh synthesis or its release in cholinergic neurons that have been stimulated by increased histamine derived from over-activated histaminergic terminals through the antagonistic action of H3R, rather than by increasing synaptic ACh via a reduction in enzymatic degradation by AChE.

Activation of H3Rs mediates a series of intracellular signaling pathways that are involved in the pathogenesis of AD, including the Gα_i/o_-protein-coupled inhibition of AC (Lovenberg et al., 1999), downstream exaggeration of cognitive decline through transduction of cAMP/CREB (Moreno et al., 2011), and activation of PI3K/AKT/GSK3β signaling (Rapanelli et al., 2016). Constitutively active H3R inhibits cAMP increase, which would otherwise activate PKA phosphorylation of CREB (Shi et al., 2012), an important signaling molecule located in the cell nucleus and responsible for the synaptic plasticity implicated in the cognitive function. In agreement with this hypothesis, in our study, cAMP/CREB signaling was seriously weakened in Aβ-mediated Alzheimer’s pathogenesis, whereas LC1405 rescued aberrant cAMP/CREB signaling both *in vitro* and *in vivo* (Figure 3E,F, 6A,B). When APPsw cells that were pretreated with the H3R agonist (RAMH) were treated with LC1405 in the presence of copper, restoration of the cAMP/CREB signaling pathway was completely abolished (Figure 6A,B). Therefore, these results suggested that H3R inactivation of LC1405 plays a pivotal role in neuronal transduction of the cAMP/CREB pathway in response to Aβ neurotoxicity. It is reasonable to believe that the ability of LC1405, as a H3R antagonist, to stimulate the release of histamine and ACh might lead to specific cellular signaling events as a property of auto-receptors in the phosphorylation-activation of CREB that contributed to enhanced cognitive function.

Besides H3R-mediated signaling through Gα_i/o_-proteins, Gβγ-subunits are known to activate specific signal transduction pathways involving the PI3K/AKT/GSK3β cascade (Rapanelli et al., 2016). Similar to other G protein-coupled receptor (GPCR) signaling transduction, H3R activation results in AKT phosphorylation at Ser 473 and subsequently GSK3β hyperphosphorylation occurring from increased phosphorylation of S9 through PI3K activation via the Gβγ-subunits of Gα_i/o_ proteins, previously demonstrated in a neuroblastoma cell line, primary cultures of cortical neurons, and in striatal slices of Sprague-Dawley rats (Bongers et al., 2007). Several studies support the findings that H3R antagonists function as indirect inhibitors of GSK3β, thereby resulting in decreased S9 phosphorylation and exert therapeutic effects in neurodegenerative disorders beyond amelioration of symptoms (Vohora and Bhowmik, 2012). Consistent with these studies, our results showed that the PI3K/AKT/GSK3β pathway was one pathway involved in LC1405 neuroprotection, which could be diminished by a H3R agonist (Figure 3G,H, 6C–E). In this regard, H3R-mediated postsynaptic PI3K/AKT/GSK3β cascade resulted involved in LC1405-induced neuroprotection against Aβ neurotoxicity.

Prior to the identification of the anti-AD effects of LC1405, the safe dosage of LC1405 was evaluated from 0.3 to 10.0 μM *in vitro* and long-term oral administration at 3 mg/kg *in vivo* (Figure 2B–G, 4A). The phenomenon that LC1405 alone activated cAMP/CREB cascade was in accordance to the fact that LC1405 targets H3Rs with the specific GPCR characteristics (Figure 3E,F). Since PI3K/AKT/GSK3β signaling pathway was regulated by several factors, we concluded that in a physiological state, a single factor mediated by LC1405 influencing PI3K/AKT/GSK3β pathway resulted in well-balanced effects. Therefore, LC1405 could trigger PI3K/AKT/GSK3β pathway and attenuate cognition deficits under physiological, but not pathological, conditions.

**FIGURE 7:**
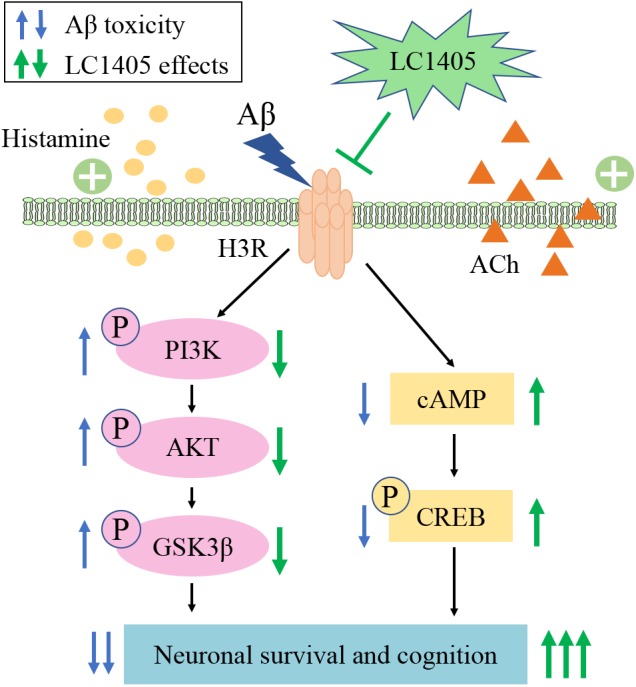
Schematic representation of LC1405 neuroprotective pathway against amyloid-β peptide (Aβ)-induced toxicity. Aβ, β-amyloid; ACh, acetylcholine; AKT, protein kinase B; cAMP, cyclic adenosine monophosphate; CREB, cAMP response element binding protein; GSK3β, glycogen synthase kinase 3β; H3R, histamine H3 receptor; PI3K, phosphatidylinositol-3-kinase.

In summary, LC1405 had beneficial effects on blocking Aβ-induced neuronal stress both *in vitro* and *in vivo*. Anti-H3R therapy using LC1405 did not affect Aβ burden or fully alter the redox balance system, but was effective in increasing the level of histamine and ACh in the brain. LC1405 improved cognition through H3R-dependent cellular signaling cascades involving cAMP/CREB and AKT/GSK3β pathways (Figure 7). Taken together, our results demonstrated that LC1405 ameliorated cognitive deficits by blocking H3R-modulated signaling transduction against Aβ-induced cellular stress. Thus, LC1405 may be a prospective H3R antagonist that holds a potential in controlling disease progression in AD patients, who already developed cognitive deficits with H3R-related ACh neurotransmission abnormalities.

## Ethics Statement

All of the protocols dealing with the maintenance and handling of animals were followed as stated in the Guidelines of the Institute of Medicinal Biotechnology, Chinese Academy of Medical Sciences.

## Author Contributions

LW performed the *in vivo* experiments. JF, HJ, QW, and SX performed the *in vitro* experiments. JF performed the *in silico* experiments. LW and JF employed statistical analysis and wrote the manuscript. RL and ZL performed principal investigation, and revised and edited the manuscript. All authors read and approved the final manuscript.

## Conflict of Interest Statement

The authors declare that the research was conducted in the absence of any commercial or financial relationships that could be construed as a potential conflict of interest.
