# Supplementary material for: 7-Pyrrolidinethoxy-4′-Methoxyisoflavone Prevents Amyloid β–Induced Injury by Regulating Histamine H3 Receptor-Mediated cAMP/CREB and AKT/GSK3β Pathways
Source: Front Neurosci. 2019 Apr 10;13:334. doi: 10.3389/fnins.2019.00334 (PMC6468582; doi:10.3389/fnins.2019.00334)
Supplement: Supplementary file 1 [file Presentation_1.pdf]

Supplementary Material

1 Materials and Methods

1.1 Strategy for the discovery of novel H3R antagonists

During the initial research period, we aimed to establish a rational screening method to identify novel lead candidates of H3R antagonists. The workflow of the study comprised three consecutive stages. Initially, the Bayesian model was used to predict novel disease-modulating compounds in a small molecule library for H3R ligands. And then, potential H3R-targeting compounds were evaluated for affinities for different human histamine receptors and different species variants of H3Rs. Next, the selected potential H3R antagonists were evaluated for their protective effect in Aβ-induced cellular stress in neuronal cells *in vitro.*

1.2 *In silico* prediction of H3R ligands using naive Bayesian classification approaches

To build classification models to discriminate H3R ligands from non-ligands, the naive Bayesian approach was used as described in our previous study (Fang et al., 2015). Briefly, after removal of duplicate molecules, a total of 2,932 H3R ligands, with an IC_{50} < 10 μM, were collected from the BindingDB database (Liu et al., 2007), and regarded positive data. In addition, a dataset of 8,796 decoy compounds was randomly extracted from the H3R decoy database, which was generated in the online database of useful decoys (DUD) (Mysinger et al., 2012) with known H3R ligands. Each compound was represented by three combinations of molecular descriptors to develop three classifiers using the Discovery Studio 2.5 software (**Supplemental Table 1**). The quality of the Bayesian classifiers was determined by the quantity of true positives, true negatives, false positives, false negatives, sensitivity, specificity, the overall prediction accuracy, and the Matthews correlation coefficient, which were given by a series of equations (Fang et al., 2015).

To identify H3R ligands, our in-house database, including 29170 compounds, was used as the screening library. Each database was processed by removing the inorganic counter ions, adding hydrogen atoms, deprotonating strong acids, protonating strong bases, generating stereo isomers and valid single 3D conformers by means of washing and minimizing energy in the molecular operating environment (MOE).

1.3 Radioligand binding assays

This study was performed to validate the affinity of H3R of predicted H3R compounds using the cell-based H3R antagonism assay. The substituted pyridazinone analogs were tested using *in vitro* binding assays by displacement of [^{3}H] N^α-methylhistamine in membranes isolated from HEK-293 cells that were transfected with cloned human H3R (hH3R) that was reported previously (Kottke et al., 2011). Briefly, HEK-293 cells stably expressing recombinant hH3R were harvested, centrifuged and homogenized in ice-cold binding buffer (50 mmol/L Na_2HPO_4/KH_2PO_4, pH 7.5). The homogenate was centrifuged (23,000 g, 30 min, 4°C) and the pellet was resuspended in binding buffer to constitute the membrane preparation that was used for the binding assay. Incubations of the membrane suspension were performed for 90 min at 25°C with either [^{3}H] N^α-methylhistamine (5 nM) alone, or a combination with different concentrations of the test compounds (seven concentrations between 0.1 nM and 100

μM). Non-specific binding was determined in the presence of the selective H3R antagonist pitolisant (10 nM), and any bound radioligand was separated from free radioligand by filtration through glass microfiber filters (GF/B Whatman, Clifton, NJ, USA). Unbound radioligand was removed by three washing steps using ice-cold medium (50 mM Tris-HCl, 120 mM NaCl, pH 7.4). The data were analyzed by Hill transformation and *K*i values were determined by the Cheng–Prusoff equation (Cheng and Prusoff, 1973). The binding analysis for mouse and rat H3Rs were performed as described above for hH3R. The binding analysis of $[^3H]$ mepyramine, $[^{125}I]$ iodoaminopotentidine and $[^3H]$ histamine binding to H1, H2 and H4 receptors, respectively, was performed according to previous reports (Bakker et al., 2004; Łażewska et al., 2014).

1.4 Screening for H3R antagonists in a cell-based assay

The cell-based H3R antagonism assay was performed by using U2OS cells that stably contain the human H3R that was linked to a preliminarily integrated beta-arrestin/TEV protease fusion protein and the beta-lactamase (bla) reporter gene under control of an UAS response element (Invitrogen, Carlsbad, CA, USA). H3R-U2OS cells were cultured in FreeStyle™ 293 Expression Medium (Invitrogen, Carlsbad, CA, USA) and plated at 7500 cells/well in a 384-well format and incubated for 18 h at 37°C. Cells were incubated with the test compounds or thioperamide (Sigma-Aldrich, St. Louis, MO, USA), which served as a positive control for 30 min at 37°C. Then, cells were stimulated with 400 nM methylhistamine (Sigma-Aldrich, St. Louis, MO, USA) for 5 h at 37°C. Assays were performed in triplicate using seven test compound concentrations between 0.01 nM and 100 μM. Cells were loaded with LiveBLAzer™-FRET B/G substrate (Invitrogen, Carlsbad, CA, USA) for 2 h prior to the assay. Fluorescence emission values at 460 nm and 530 nm were obtained using a Spark 20M multimode microplate reader (Tecan Group Ltd., Mannedorf, Switzerland), and the percentage of inhibition was plotted against the indicated concentrations of the test compound. IC_{50} values were graphically determined from log concentration–inhibition curves.

1.5 cAMP accumulation assay

Intracellular cAMP accumulation was measured using HEK293 cells expressing hH3R, co-treated with forskolin, (*R*)-(−)-*α*-methylhistamine (RAMH) and LC1405. Briefly, forskolin (10 μM), RAMH (15 nM) and LC1405 (ranging from 0.1 nM-100 μM) were added simultaneously to cell suspension. Cells stimulation was performed for 30 min at room temperature. The cells were treated with lysis buffer, and then 10 μL of the lysate was tested for measurement of cAMP concentration. cAMP accumulation levels were determined using a cAMP assay kit (R&D Systems, Minneapolis, MN, USA). The optical density at 570 nm was read on a Spark 20M multimode microplate reader (Tecan Group Ltd., Mannedorf, Switzerland). Measured OD signal was translated into actual quantities of produced cAMP on the basis of cAMP standard curve.

1.6 Primary neuronal cell culture and treatments

Primary cortical neuron cultures were prepared as described previously with minor modifications (Liu et al., 2007). Primary cultures of cortical neurons were prepared from Sprague Dawley rat embryos. In brief, the brain cortex was dissected, the cerebral vascular membrane was stripped of, digested with 0.125% trypsin, and dispersed by repeating aspirations through a Pasteur pipette following centrifugation. Cortical neurons were cultured in Neurobasal medium (Invitrogen, Carlsbad, CA, USA), supplemented with B27 (Invitrogen, Carlsbad, CA, USA) and 0.5 mM glutamine and were maintained in a humidified incubator at 37°C with 5% CO_2/95% air. Half the volume of medium was removed and

replaced every 3 days. Eight days after the initial plating, primary cortical neurons were used for experiments. After initiating aggregated $A\beta_{25\text{-}35}$ or fibrillar β-amyloid 1-40 ($fA\beta_{1\text{-}40}$)-induced injury, primary cortical neurons were treated with different concentrations of LC1405 and neurons were incubated for 72 h. In $A\beta_{25\text{-}35}$- and $fA\beta_{1\text{-}40}$-induced toxicity models, neurons were randomly divided into two groups, one group was supplemented with 10 μM $A\beta_{25\text{-}35}$ or 20 μM $fA\beta_{1\text{-}40}$, whereas the other group was not supplemented with $A\beta_{25\text{-}35}$ or $fA\beta_{1\text{-}40}$. Neurons in each group were further divided into subgroups based on LC1405 concentrations: 0.3 μM, 1.0 μM and 3.0 μM.

1.7 The construction of APPsw cells

SH-SY5Y cells were cultured in DMEM medium supplemented with fetal bovine serum. The APP adenovirus vector was concocted by Syngentech company (Beijing, China). SH-SY5Y cell were planted in 6-well plate and the APP adenovirus vector were infected into cells. Then, after selected with puromycin, we acquired a monoclonal of cells stably expressed Swedish mutant form of human APP.

1.8 Western blot analysis

The mouse cortex was homogenized in RIPA buffer (Cell Signaling Technology, Danvers, MA, USA) containing protease inhibitor, phosphatase inhibitor, and phenylmethyl sulfonylfluoride (PMSF). Forty μg protein per lane were run on polyacrylamide gel, transferred onto a polyvinylidenedifluoride membrane, blocked with 5% BSA in Tris-buffer saline containing 0.1% Tween-20 (TBST) for 2 h, and subsequently incubated with the primary antibody overnight, using the antibodies diluted in blocking solution as follows: rabbit polyclonal anti-p-PI3K p85α (Y607) (1 : 500, Abcam), rabbit polyclonal anti-PI3K p85 (1 : 500, Abcam), rabbit polyclonal anti-p-AKT (Ser473) (1:500, CST), rabbit polyclonal anti-AKT (1 : 500, CST), rabbit polyclonal anti-p-GSK3β (Ser9) (1 : 500, CST), rabbit polyclonal anti-GSK3β (1 : 500, CST), and mouse monoclonal anti-β-actin (1 : 10000, CST). Membranes were washed with TBST prior to incubation with horseradish peroxidase-labeled (HRP)-linked secondary antibody (1 : 1000, ZSGB-Bio, Beijing, China) at room temperature for 1 h. The signals were detected using an enhanced chemiluminescence kit. Chemiluminescence image acquisition and densitometric band quantitation were performed using Fusion-FX6 imaging system (Vilber Lourmat, Marne-la-Valle, France).

1.9 Statistical analysis

Data are represented as mean ± SEM. $p < 0.05$ was considered statistically significant. Statistical analyses were performed using SPSS software (SPSS, Inc., Chicago, IL, USA). Comparisons were performed using one-way ANOVA, followed by Tukey's post-hoc testing to analyze the differences between groups.

2 Results

2.1 The performance of three classifiers and *in silico* prediction of H3R ligands

Among the three generated classifiers, the best naïve Bayesian (NB) model, named as the M3 model, obtained the highest performance based on 16 descriptors (15 descriptors filtered and ECFP_6), which resulted in Matthews correlation coefficients of 0.990 and 0.994 for the training and test set, respectively. Moreover, the M3 classifier identified important fragments in two classifying groups (positive and negative groups) for Histamine H3, providing 20 positive fragments with favorable

binding (**Supplementary Fig. 1A**), 20 negative fragments (**Supplementary Fig. 1B**), and corresponding Bayesian scores. To identify H3R lead ligands using the M3 model in drug discovery research, we performed a virtual screening using our in-house database (including 29170 compounds). Using the M3 model, 1273 compounds were identified as potential H3R ligands, among which LC1405 (7-pyrrolidinethoxy-4'-methoxyisoflavone), having a molecular weight of 381.43 Da, got a favorable score of 0.64 (**Supplementary Table 2**).

2.2 *In vitro* profile of LC1405 in binding and functional assays at the histamine receptors

We evaluated the molecular and cellular action of LC1405 on the human histamine receptor subtypes. As shown in **Supplementary Table 2**, LC1405 demonstrated a good affinity for hH3Rs in HEK293 cells (65.50 ± 4.48 nM), illustrated a strong activity of H3R antagonism (63.04 ±2.83 nM) and blocked the decrease of cAMP induced by RAMH (158.99 ± 7.95 nM). In addition, LC1405 had weak affinities for other histamine receptors. These results suggested that LC1405 was classified as an antagonist at H3 receptors.

2.3 LC1405 illustrated neuroprotective effects against $A\beta_{25-35}$ and $fA\beta_{1-40}$-induced toxicity *in vitro*

A cytotoxicity assay was performed to evaluate the neuroprotective effects of LC1405 on rat primary cortical neurons against $A\beta_{25-35}$- and $fA\beta_{1-40}$-induced toxicity. Cell viability was significantly decreased to 58.02 ± 2.64% and 71.01 ± 2.91% in neurons that were exposed to 10 μM $A\beta_{25-35}$ and 20 μM $fA\beta_{1-40}$, respectively (**Supplementary Fig. 2B-C,** both $p < 0.001$ versus control). Treatment with LC1405 at concentrations of 0.3 μM, 1.0 μM, and 3.0 μM, after exposure to 10 μM $A\beta_{25-35}$ or 20 μM $fA\beta_{1-40}$ increased cell viability in a concentration-dependent manner ($A\beta_{25-35}$: 71.05 ± 2.28%, 86.06 ± 4.78%, 89.49 ± 4.36% *vs.* 58.02 ± 2.64%; $fA\beta_{1-40}$: 80.65 ± 1.51%, 86.43 ± 1.39%, 91.52 ± 1.89% *vs.* 71.01 ± 2.91%; $p < 0.05$, $p < 0.001$). LC1405 treatment did not have significant effects on neurons that were not exposed to $A\beta_{25-35}$ or $fA\beta_{1-40}$ treatment at any of the concentrations tested.

2.4 LC1405 attenuated the expressions of phosphorylated PI3K, AKT, and GSK3β in APP/PS1 mouse cortex

Western blot analysis showed a marked phosphorylated enhancement of PI3K at Y607, AKT at Ser473, and GSK3β at Ser9 by 1.47, 1.57, and 1.93 fold (**Supplementary Fig. 3**, all $p < 0.01$), respectively, in the cortex of APP/PS1 mice compared to WT controls. In contrast, LC1405 treatment reduced the phosphorylated PI3K, AKT, and GSK3β at in the cortex of APP/PS1 mice compare to APP/PS1 control mice (p-PI3K/PI3K: 1.23 ± 0.03 *vs.* 1.47 ± 0.06; p-AKT/AKT: 1.21 ± 0.05 *vs.* 1.57 ± 0.07; p-GSK3β/ GSK3β: 1.57 ± 0.04 *vs.* 1.93 ± 0.11; all $p < 0.05$). These results suggested that LC1405 treatment could attenuate the activation of PI3K/AKT/GSK-3β signal transduction in APP/PS1 mouse cortex.

2.5 Expression of Swedish mutant form of human APP were increased in monoclonal APPsw cells.

Supplementary Fig. 4A showed the information of the plasmid of Swedish mutant form of human APP(APPsw). We detected the expression of flag protein which were co-translocation with APPsw and found that monoclonal APPsw cells significantly increased APP695 expression compared to primary infection cells (**Supplementary Fig. 4B**).

3 References

Liu, T., Lin, Y., Wen, X., Jorissen, R.N., Gilson, M.K. (2007). BindingDB: a web-accessible database of experimentally determined protein-ligand binding affinities. Nucleic Acids Res. 35, D198-201. doi: 10.1093/nar/gkl999

Mysinger, M.M., Carchia, M., Irwin, J.J., Shoichet, B.K. (2012). Directory of useful decoys, enhanced (DUD-E): better ligands and decoys for better benchmarking. J. Med. Chem. 55, 6582-6594. doi: 10.1021/jm300687e

Fang, J., Li, Y., Liu, R., Pang, X., Li, C., Yang, R., et al. (2015). Discovery of multitarget-directed ligands against Alzheimer's disease through systematic prediction of chemical-protein interactions. J. Chem. Inf. Model 55, 149-164. doi: 10.1021/cj500574n

Kottke, T., Sander, K., Weizel, L., Schneider, E.H., Seifert, R., Stark, H. (2011). Receptor-specific functional efficacies of alkyl imidazoles as dual histamine H3/H4 receptor ligands. Eur. J. Pharmacol. 654, 200-208. doi: 10.1016/j.ejphar.2010.12.033

Cheng, Y. and Prusoff, W.H. (1973). Relationship between the inhibition constant (K1) and the concentration of inhibitor which causes 50 per cent inhibition (I50) of an enzymatic reaction. Biochem. Pharmacol. 22, 3099-108.

Bakker, R.A., Weiner, D.M., ter Laak, T., Beuming, T., Zuiderveld, O.P., Edelbroek, M., et al. (2004) 8R-lisuride is a potent stereospecific histamine H1-receptor partial agonist. Mol. Pharmacol. 65, 538-49. doi: 10.1124/mol.65.3.538

Łażewska, D., Więcek, M., Ner, J., Kamińska, K., Kottke, T., Schwed, J.S., et al. (2014). Aryl-1,3,5-triazine derivatives as histamine H4 receptor ligands. Eur. J. Med. Chem. 83, 534-46. doi: 10.1016/j.ejmech.2014.06.032

Liu, R., Wei, X.B., Zhang, X.M. (2007). Effects of acetylpuerarin on hippocampal neurons and intracellular free calcium subjected to oxygen-glucose deprivation/reperfusion in primary culture. Brain Res. 1147, 95-104. doi: 10.1016/j.brainres.2007.01.146

4 Supplementary Figures

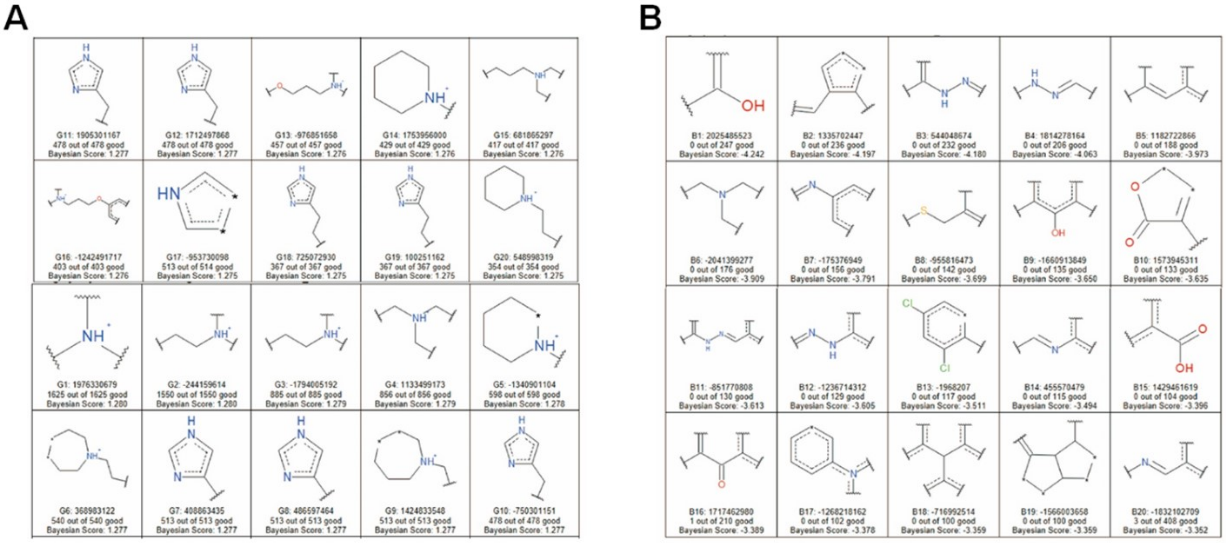

Supplementary Figure 1. Analysis of the important fragments given by a naive Bayesian classifier. (A) Molecular fragments of Histamine H3 subtype ligands showing a positive contribution. (B) Molecular fragments Histamine H3 subtype ligands showing a negative contribution.

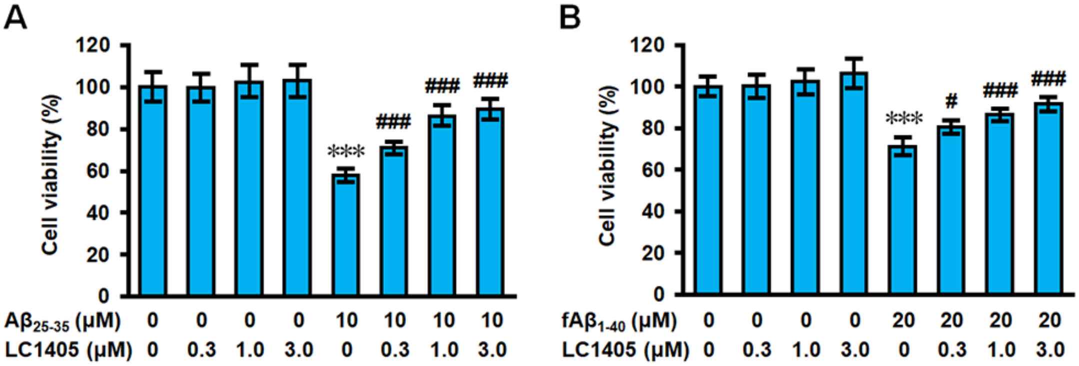

Supplementary Figure 2. LC1405 treatment increased cell viability after exposure to: (A) 10 μM $A\beta_{25-35}$ or (B) 20 μM $fA\beta_{1-40}$. Data are expressed as mean ± SEM. $n = 8$. ***$p < 0.001$ *vs.* control, $^{\#}p < 0.05$, $^{\#\#\#}p < 0.001$ *vs.* $A\beta_{25-35}$ or $fA\beta_{1-40}$.

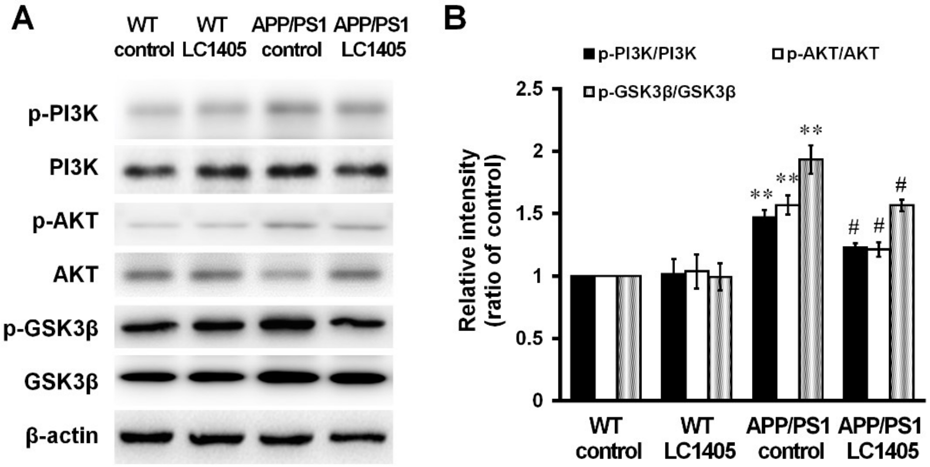

Supplementary Figure 3. LC1405 treatment inhibited PI3K/AKT/GSK3β signal pathway in APP/PS1 mouse cortex. (A) Representative Western blots of phosphorylated PI3K at Y607, AKT at Ser473, and GSK3β at Ser9 in the cortex of APP/PS1 mice. (B) Quantified results were normalized to β-actin expression. Data are expressed as mean ± SEM. $n = 4$. $^{**}p < 0.01$ *vs.* WT control mice, $^{\#}p < 0.05$ *vs.* APP/PS1 control mice.

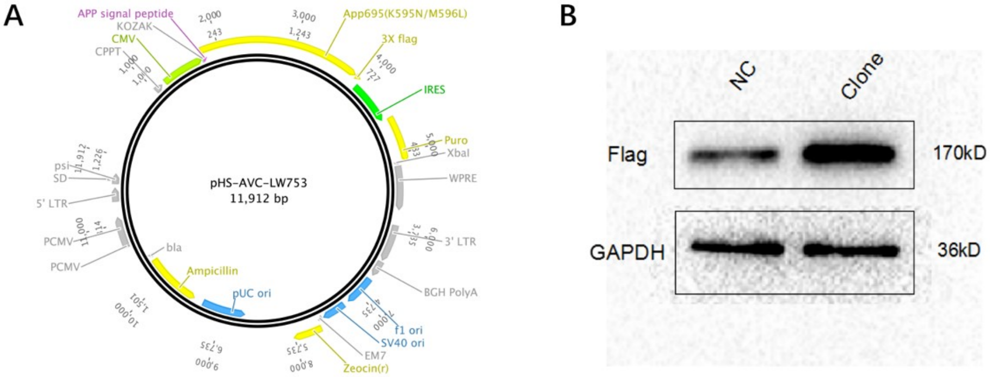

Supplementary Figure 4. (A) APPsw plasmid circle map. (B) APP expression significantly increased in APPsw cells.

Supplementary Tables

Supplementary Table 1. Molecular descriptors used in this study.

Descriptor class	Number of descriptors	Descriptors
Combination 1	8	ALogP, Molecular_Weight, Num_Aromatic Rings, Num_H_Acceptors, Num_H_Donors, Num_Rings

		Num_RotatableBonds, Molecular_Fractional Polar Surface Area
Combination 2	15	ALogP98；Molecular_Solubility；JX; Kappa_3_AM; ES_Sum_ssNH; Num_H_Acceptors; Num_H_Donors_Lipinski; SAscore_Fragments; QED_ALOGP; ES_Sum_dsCH; CHI_V_2; ES_Sum_sssCH; ES_Sum_dsN; Molecular_Fractional PolarSASA; QED_AROM.
Combination 3	16	Combination 2 + ECFP6

Supplementary Table 2. Bayesian score and pharmacological profiles of LC1405 in binding and functional assays at human histamine receptors.

EstPGood	Affinity at human histamine receptors (*K*i)				Functional properties at histamine H3 receptor	
	**H1 (nM)**	**H2 (nM)**	**H3 (nM)**	**H4 (nM)**	**Cell-based H3R antagonism assay (IC50, nM)**	**cAMP accumulation assay (EC50, nM)**
0.64	3059.38±233.07	12267.78±1283.76	65.50±4.48	34461.18±1952.90	63.04±2.83	158.99±7.95

Notes: EstPGood, the expected normal distribution of scores in the active and inactive class and a normalized score that is comparable across models. IC50, the effectiveness in inhibiting H3R activity. *K*i, inhibitory constant of human histamine receptor binding in the corresponding subtype transfected HEK-293 cells. Data are expressed as mean ± SEM. *n* = 3–5 independent experiments per group.
